# Altered expression pattern of integrin alphavbeta3 correlates with actin cytoskeleton in primary cultures of human breast cancer

**DOI:** 10.1186/1475-2867-7-16

**Published:** 2007-10-02

**Authors:** Sophia Havaki, Mirsini Kouloukoussa, Kawther Amawi, Yiannis Drosos, Leonidas D Arvanitis, Nikos Goutas, Dimitrios Vlachodimitropoulos, Stamatis D Vassilaros, Eleni Z Katsantoni, Irene Voloudakis-Baltatzis, Vassiliki Aleporou-Marinou, Christos Kittas, Evangelos Marinos

**Affiliations:** 1 Laboratory of Histology and Embryology, Medical School, University of Athens, 75 Mikras Asias Str., 11527 Goudi, Greece; 2 Department of Genetics and Biotechnology, School of Biology, University of Athens, Panepistimioupoli, 15701 Ilissia, Greece; 3 Department of Anatomy and Pathology, Medical School, University of Thessaly, 22 Papakiriazi Str., 41222, Larissa, Greece; 4 Laboratory of Forensic Medicine and Toxicology, Medical School, University of Athens, 75 Mikras Asias Str., 11527 Goudi, Greece; 5 Prolipsis" Medical Centre, 3 Sevastias Str., Athens, Greece; 6 Hematology Division, Biomedical Research Foundation, Academy of Athens, 4 Soranou Ephesiou, 11527 Athens, Greece; 7 Department of Electron Microscopy and Cell Biology, Research Centre of Oncology "G. Papanikolaou", Saint Savvas Anticancer Hospital, Alexandras Av. 171, Athens, Greece

## Abstract

**Background:**

Integrins are transmembrane adhesion receptors that provide the physical link between the actin cytoskeleton and the extracellular matrix. It has been well established that integrins play a major role in various cancer stages, such as tumor growth, progression, invasion and metastasis. In breast cancer, integrin alphavbeta3 has been associated with high malignant potential in cancer cells, signaling the onset of widespread metastasis. Many preclinical breast cancer studies are based on established cell lines, which may not represent the cell behavior and phenotype of the primary tumor of origin, due to undergone genotypic and phenotypic changes. In the present study, short-term primary breast cancer cell cultures were developed. Integrin alphavbeta3 localization was studied in correlation with F-actin cytoskeleton by means of immunofluorescence and immunogold ultrastructural localization. Integrin fluorescence intensities were semi-quantitatively assessed by means of computerized image analysis, while integrin and actin expression was evaluated by Western immunoblotting.

**Results:**

In the primary breast cancer epithelial cells integrin alphavbeta3 immunofluorescence was observed in the marginal cytoplasmic area, whereas in the primary normal breast epithelial cells it was observed in the main cell body, i.e. in the ventrally located perinuclear area. In the former, F-actin cytoskeleton appeared well-formed, consisting of numerous and thicker stress fibers, compared to normal epithelial cells. Furthermore, electron microscopy showed increased integrin alphavbeta3 immunogold localization in epithelial breast cancer cells over the area of stress fibers at the basal cell surface. These findings were verified with Western immunoblotting by the higher expression of integrin beta3 subunit and actin in primary breast cancer cells, revealing their reciprocal relation, in response to the higher motility requirements, determined by the malignant potential of the breast cancer cells.

**Conclusion:**

A model system of primary breast cancer cell cultures was developed, in an effort to maintain the closest resembling environment to the tumor of origin. Using the above system model as an experimental tool the study of breast tumor cell behavior is possible concerning the adhesion capacity and the migrating potential of these cells, as defined by the integrin alphavbeta3 distribution in correlation with F-actin cytoskeleton.

## Background

Integrins are a family of glycosylated, heterodimeric transmembrane adhesion receptors that mediate cellular attachment to the extracellular matrix and to adjacent cells [1]. They have a widespread distribution in cells and tissues, as they are involved in morphogenetic cell movements and migration, such as in gastrulation, neurulation and histogenesis [2], as well as in inflammation, wound healing and thrombotic events [3,4]. Upon cellular interaction with extracellular matrix, integrin receptors function as bidirectional transducers of extra- and intracellular signals implicating in the regulation of immediate gene expression, cell proliferation, differentiation, survival/anoikis and angiogenesis [5-8].

Furthermore, it is well established that integrins, due to aberrant adhesive events and cellular signals that alter gene expression and influence cell survival, contribute to various cancer stages, such as malignant transformation, tumor growth and progression, invasion and metastasis [9-11]. In cancer growth, both quantitative and qualitative alterations in integrin expression have been observed. Some integrins are overexpressed or no longer expressed, while others become phosphorylated, affecting their cytoskeletal and extracellular ligand binding properties [12].

Integrin alphavbeta3 – a vitronectin receptor – has been implicated in the pathophysiology and progression of several malignant tumors, such as melanoma [13,14], glioma [15], ovarian [16], prostate [17] and breast cancer [18,19]. Specifically, nearly in all breast cancer tumors with a metastasis to bone, integrin alphavbeta3 was highly expressed [20]. In vitro studies of breast cancer cultures have supported the positive correlation of the increased expression of alphavbeta3 with the ability of the cancer cells to adhere to extracellular matrix, to migrate, to regulate protease maturation [21], as well as to interact and form aggregates with platelets, contributing to breast tumor cell adhesion to the subendothelial matrix under dynamic blood flow conditions [22,23]. Thus, the presence of integrin alphavbeta3 on breast cancer tumors signals the onset of widespread metastasis [11,24].

Integrins, being cell-substrate adhesion molecules, provide the physical and essential link between the actin cytoskeleton and the extracellular matrix during cell migration [25,26]. This connection is dynamically reorganized in response to mechanical, chemokine and growth factor signals, resulting in the continuous growth and reorganization of actin filaments in protruding organelles at the front of migrating cells, such as filopodia and lamellipodia, and in parallel, in the controlled retraction of adhesive contacts at the rear of these cells [27-29]. The regions of the plasma membrane where integrins connect actin cytoskeleton to the extracellular matrix, through various structural and regulatory adaptor proteins, are specialized adhesive structures called *focal adhesions *or *focal contacts *[30]. At focal adhesions, actin filaments are anchored in the form of bundles, termed stress fibers.

Ligand binding to integrins leads to integrin clustering and recruitment of actin filaments and signaling proteins to the cytoplasmic domain of integrins [31]. Concerning the integrin alphavbeta3, the focal adhesion formation begins upon the presence of the extracellular ligand that triggers the activation and clustering of the integrin.

Given that the behavior of any given tumor is a complex interaction between tumor cells and the host environment, cell culture models are the first step toward characterizing molecules that influence tumor cell behavior in vivo [24]. Established breast cancer cell lines have been widely used to provide meaningful data for evaluating the pathobiology of this kind of cancer, as the cultured cells are easy to handle and can be grown in almost infinite quantities with a high degree of homogeneity. However, there is a fundamental disadvantage: Cell lines are prone to genotypic and phenotypic alterations during their continual culture, resulting in a drift away from the phenotype of the originating tumor in vivo. On the contrary, primary cultures derived directly from tumors have the advantage that the cultured cells are directly isolated from the tumor site and their phenotype closely represents the pathobiology of the original tumor in vivo, as cancer cells are cultured for a finite length of time and they have little opportunity to undergo genotypic transformations [32]. Although there is correlative evidence supporting the role of tumor-specific alphavbeta3 expression in breast cancer progression [20], its precise contribution to primary tumor growth and metastasis is still unclear [11].

In the present work, for the first time, the correlation of integrin alphavbeta3 expression with actin cytoskeleton distribution was studied in primary cell cultures of human breast cancer biopsies using immunofluorescent localization of integrin alphavbeta3 combined with phalloidin-rhodamine fluorescence for F-actin visualization. The relative integrin alphavbeta3 fluorescence intensities at the periphery and the main body of the cancer cells were semi-quantitatively assessed with the aid of a computerized image analysis system. A qualitative evaluation of integrin alphavbeta3 localization in correlation with the distribution of F-actin bundles in breast cancer cells, was also undertaken ultrastructurally, while quantification of integrin and actin expression was evaluated by Western immunoblotting. For comparison, the same procedure was carried out in primary cultures of breast cells developed from biopsies of normal tissues adjacent to the tumor. The results showed a shift of integrin alphavbeta3 immunofluorescence from the main cell body – particularly at the perinuclear area, near the ventral cell surface – in the primary normal breast epithelial cells to the marginal area in the primary breast cancer epithelial cells. In parallel, in the latter, F-actin cytoskeleton appeared well-formed, consisting of numerous and thicker stress fibers. At the ultrastructural level, increased integrin alphavbeta3 immunogold localization was observed in epithelial breast cancer cells over the area of stress fibers at the basal cell surface. These findings were also verified with Western immunoblotting by the higher expression of integrin beta3 subunit and actin in primary breast cancer cells. Given the above, the primary breast cancer cell cultures developed without any enzymatic treatment – to retain the integrin alphavbeta3 status intact – consist a model system that can be successfully used for the study of breast tumor cell behaviour, concerning the adhesion capacity and the migrating potential of these cells.

## Results

### Scanning electron microscope

**Figure 1:**
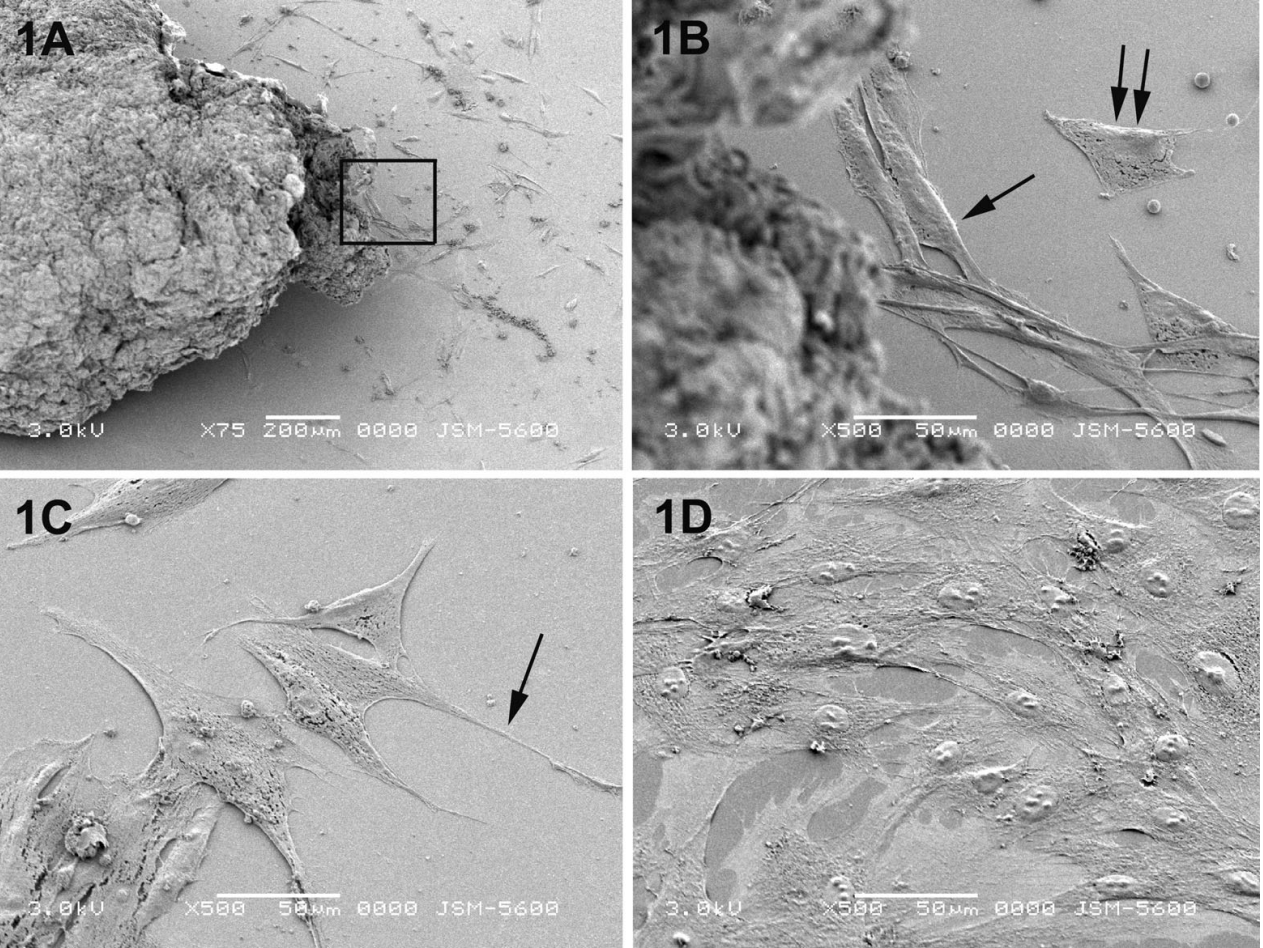
Scanning electron micrographs of primary breast cancer cells. (A) Low magnification overview showing a part of tissue fragment of the breast cancer biopsy and the cells with epithelial and fibroblast-like morphology which migrate out from it. (B) Higher magnification of the boxed area in figure 1A exhibiting the mixed cell population that has grown out from tissue fragment consisting of spindle shaped fibroblasts (arrows) and polygonal astrocytic epithelial cells (double arrows).(C) Epithelial-like cells of breast cancer primary culture appearing flat with a relatively smooth surface and well-formed cytoplasmic extensions (arrow). (D) A large number of epithelial-like breast cancer cells which are well attached to the substrate of the glass cover slip and with well developed filopodial extensions forming stratified layers. In the majority of the cells prominent nuclei with numerous nucleoli are clearly observed.

Under the scanning electron microscope, primary cell cultures developed from human breast cancer biopsies showed an effective migration of epithelial and fibroblast-like cells (Fig. 1A). The latter, consisting the minority of the liberated cells, had the typical bipolar spindle shape (Fig. 1B). The epithelial-like cells were characterized by their typical polygonal astrocytic shape (Figs. 1B, 1C) with well-developed filopodial extensions, which in some cases were quite long (Fig. 1C, arrow). Their surface topography was relatively smooth with prominent nuclei. Most of the epithelial-like cancer cells were grown independently, forming contacts with the adjacent cells via their cytoplasmic projections. These cells were flattened at the areas of filopodial extensions, being well attached to the substrate, while the rest part of the cells was elevated, indicating retraction of the substrate and a tendency of the cells to move on. In some of the cases, epithelial-like cancer cells grew in the culture medium as large groups of cells, flattened and expanding lamellipodial projections, forming stratified layers (Figure 1D).

### Correlation of integrin alphavbeta3 immunofluorescence with F-actin phalloidin-rhodamine fluorescence

**Figure 2:**
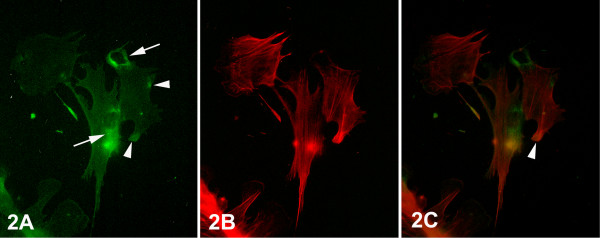
Epifluorescent micrographs of epithelial cells of primary culture of normal breast tissue. (A) In the majority of the cells at the ventral surface, intense integrin alphavbeta3 immunofluorescence was observed at their perinuclear areas (arrows), and low diffused localization in the rest of the surface. Integrin alphavbeta3 aggregations (arrowheads) at the cell periphery were scarcely observed. (B) F-actin cytoskeleton, as visualized by rhodamine-conjucated phalloidin, was characterized by thin stress fibers arranged in parallel arrays across the cells. (C) Merged images of (A) and (B) showing the colocalization of stress fibers with integrin alphavbeta3 clusters at the sites of focal contacts (arrowhead). [750×].

By means of epifluorescent microscopy, the distribution of integrin alphavbeta3 was studied in correlation with F-actin cytoskeleton in epithelial-like cells of primary cultures of breast cancer biopsies and of biopsies of normal tissues adjacent to the tumor. In most of the epithelial cells of normal tissues, the immunofluorescent staining of integrin alphavbeta3 showed a slightly diffused pattern at the ventral cell surface, but mostly an intense distribution of integrin alphavbeta3 at the perinuclear area, as deduced by the intensity of fluorescence (Fig. 2A, arrows). Furthermore, fluorescent aggregations indicating integrin clustering, were scarcely observed at the periphery of the cell (Fig. 2A, arrowheads). Fluorescence of rhodamine-conjucated phalloidin revealed a well organized actin filament cytoskeleton consisting of thin bundles of stress fibers arranged across the cell bodies in parallel arrays, while some of them were oriented towards the cytoplasmic filopodial extensions (Fig. 2B). The merged images of integrin alphavbeta3 and actin filaments fluorescence did not reveal an appreciable colocalization, except at the focal contact sites, where integrin alphavbeta3 is localized and stress fibers terminate (Fig. 2C, arrowhead).

**Figure 3:**
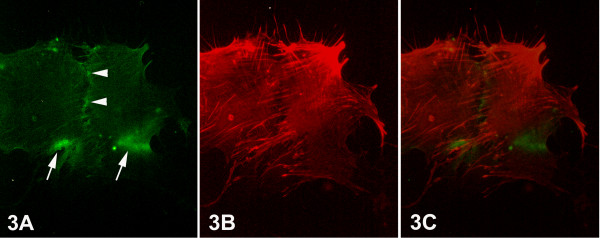
Epifluorescent micrographs of epithelial cells of primary culture of breast cancer tissue. (Figs. 3A) Bright aggregations of integrin alphavbeta3 immunofluorescence – indicating integrin clustering – were observed at the marginal areas of the ventral surface of the cells (arrows). Fine granular integrin alphavbeta3 immunofluorescence was also observed at the boundary between the cells in contact (arrowheads). (Fig. 3B) F-actin cytoskeleton, as visualized by rhodamine-conjucated phalloidin, appeared well developed, constituted by numerous thick stress fibers, which were arranged towards the integrin alphavbeta3 immunolocalizations, as demonstrated also in the merged images (Fig. 3C). [750×].

**Figure 4:**
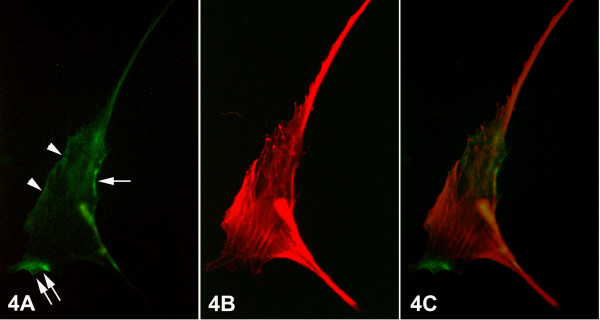
Epifluorescent micrographs of epithelial cell of primary culture of breast cancer tissue. (Fig. 4A) Bright aggregations of integrin alphavbeta3 immunofluorescence – indicating integrin clustering – were observed along the periphery of the ventral surface of the cell (arrow), while some of them were formed at the leading edge of the advancing lamellipodium (double arrows). Less bright fine granular integrin alphavbeta3 immunofluorescence was also observed along the cell surface (arrowheads). (Fig. 4B) F-actin cytoskeleton, as visualized by rhodamine-conjucated phalloidin, appeared well developed, constituted by numerous thick stress fibers, which were arranged towards the integrin alphavbeta3 immunolocalizations, as demonstrated also in the merged images (Fig. 4C). [750×]

The primary epithelial breast cancer cells appeared larger in size than the normal ones (Figs. 3, 4). The pattern of integrin alphavbeta3 immunofluorescence was mainly observed at the marginal region of the cells at the sites of focal contacts, contributing to the adherence of the cancer cells onto the substrate. Thus, integrin alphavbeta3 immunofluorescence revealed a pattern of bright aggregations distributed along the periphery of the ventral surface of the cells (Figs. 3A, 4A, arrows), or at the leading edge of the advancing lamellipodium (Fig. 4A, double arrows). In addition, integrin alphavbeta3 localization was also recorded as fine granular fluorescence dispersed along cell-cell contacts (Fig. 3A, arrowhead), or at the cell's periphery (Fig. 4A, arrowhead). F-actin cytoskeleton fluorescence in epithelial breast cancer cells showed numerous and thicker stress fibers than those observed in normal cells. When the cells were in contact, the stress fibers were oriented vertically to the boundary between them (Fig. 3B) or towards areas of integrin immunofluorescence (Fig. 4B). The merged images demonstrated the correlation of the distribution of the stress fibers with the integrin alphavbeta3 immunofluorescence, indicating that the orientation of stress fibers is determined by/depending on the formation of integrin clustering (Fig. 3C, 4C).

### Assessment of the relative integrin alphavbeta3 fluorescence intensities with image analysis morphometry

Because of the observed differential distribution of integrin alphavbeta3 immunofluorescence between the marginal area and the main cell body of the epithelial cells of the breast cancer biopsies compared with those of normal tissues, the relative integrin alphavbeta3 fluorescence intensities per cellular area between normal and cancer cells were measured as described in the "Material and Methods" using image analysis morphometry. The data were assessed with the frequency distribution test, i.e. studying the frequency of occurrence of values dividing the range of values into 8 classes. The higher the value, the higher the relative integrin alphavbeta3 fluorescence intensity is.

**Figure 5:**
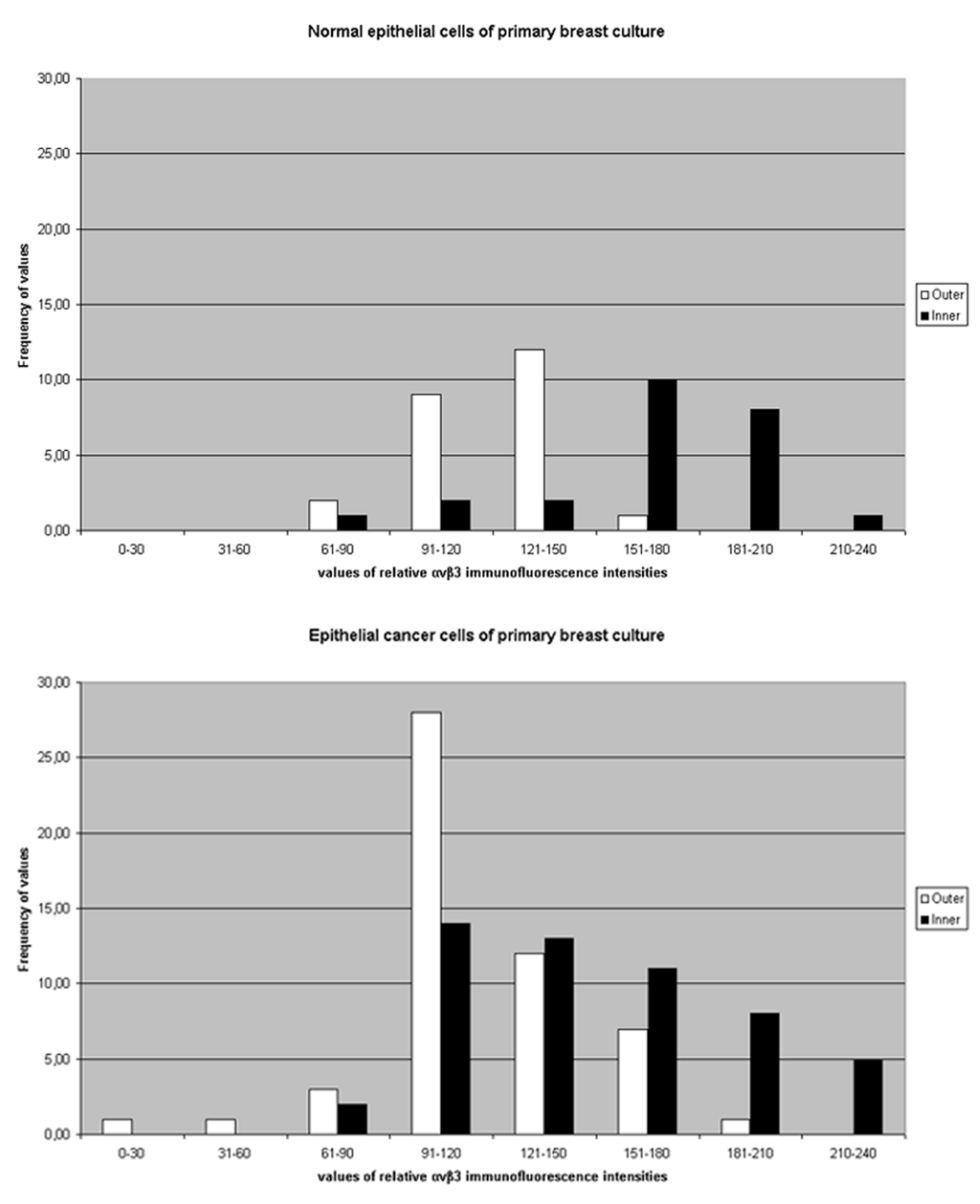
Histograms displaying the heterogeneous frequency distribution of values of relative integrin alphavbeta3 immunofluorescence intensities from epithelial cells of primary breast cultures of normal tissue (A), and from epithelial cells of primary breast cancer cultures (B). The white bars represent the frequency of values derived from the marginal areas of the cells (termed as outer on the histogram), while the black bars represent the frequency of values derived from the main cell bodies (termed as inner on the histogram).

According to the histogram of epithelial cells of primary breast cultures of normal tissues (Fig. 5A), the frequency of values derived from the main cell bodies (termed as inner on the histogram, black bars) showed a distribution with the highest frequencies belonging in two classes of high intensity values (151–180, 181–210). Very low frequency was observed in the last class representing the highest intensity values (210–240). This frequency distribution is consistent with the integrin alphavbeta3 immunofluorescence in the main cell body of the normal epithelial cells, which is restricted mainly at the ventrally located perinuclear area of the cells. On the other hand, the frequency of values derived from the marginal areas of the cells (termed as outer on the histogram, white bars) showed the highest frequency in the fifth class (121–150), representing the few integrin fluorescent aggregations formed at the cell periphery.

According to the histogram of epithelial cancer cells of primary breast cultures (Fig. 5B), intensity values derived from the main cell bodies (black bars) were distributed to the moderate and higher classes of immunofluorescence intensity. Probably, this is due to the occasional presence of integrin alphavbeta3 immunofluorescence in the main cell body. In parallel, the frequency of values derived from the marginal areas of the cells (white bars) was distributed over all of the classes of intensity, except of the last one. The highest frequency of values was observed in the classes of moderate (91–120) and higher (121–150, 151–180) intensity. From the above, it is clear that the frequency distribution of intensity is obviously different between normal and cancer cells of breast biopsies.

### Immunogold labeling of integrin alphavbeta3

To further examine the correlation of integrin alphavbeta3 expression with the actin cytoskeleton revealed by immunofluorescence, the precise pattern of integrin alphavbeta3 localization was ultrastructurally studied by means of immunogold method, in comparison with the actin cytoskeleton.

**Figure 6:**
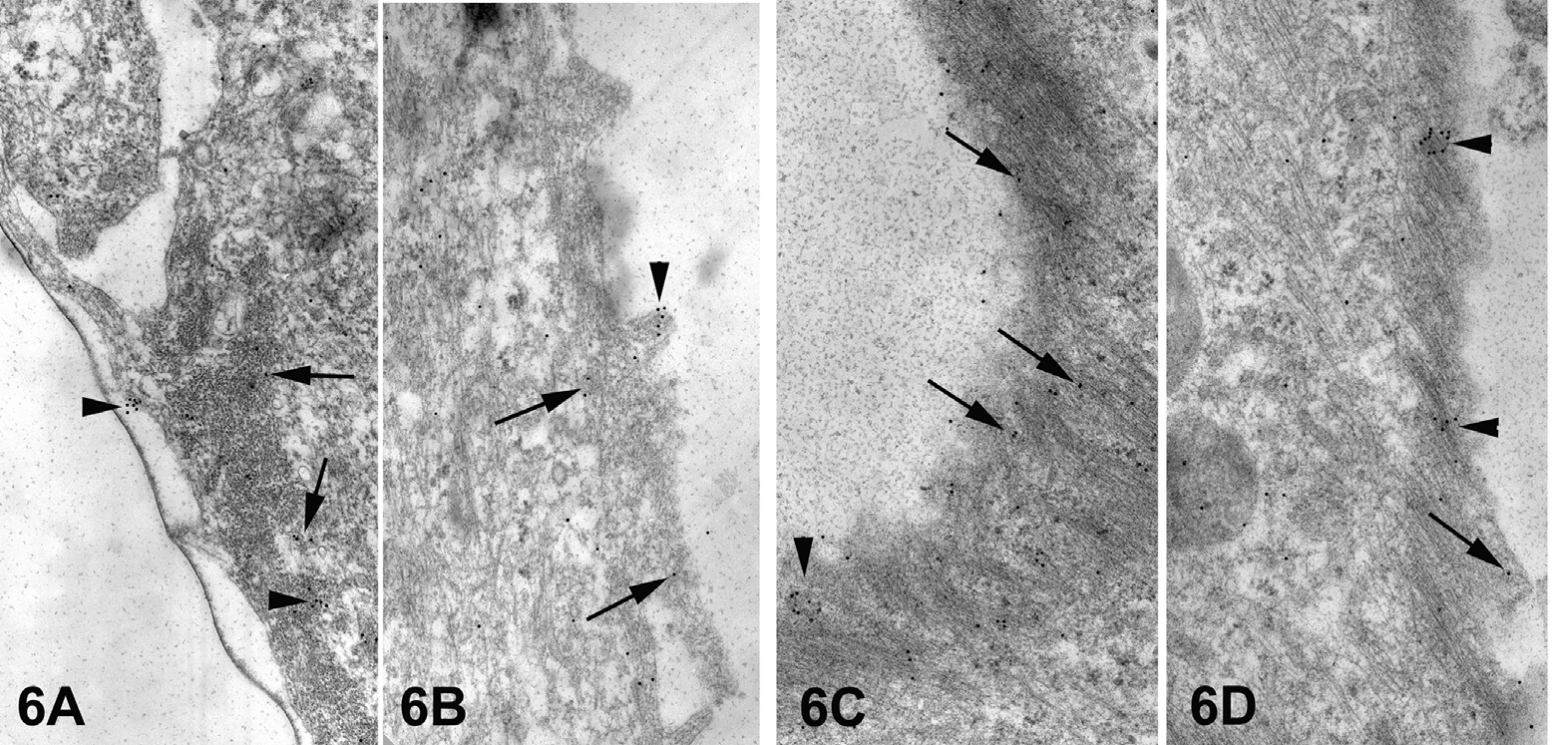
Electron micrographs showing the integrin alphavbeta3 immunogold labeling in epithelial cells of primary cultures of normal breast tissues (A, B) and of breast cancer biopsies (C, D). In both cases staining pattern is essentially the same i.e. mostly single gold particles (arrows), and occasionally aggregates (arrowheads) over the region of the stress fibers. However, in cancer epithelial cells an increased integrin alphavbeta3 immunogold labeling is observed. [25,250×].

In epithelial cells of primary breast cultures of normal tissues, integrin alphavbeta3 gold particles were dispersed heterogeneously over the area of stress fibers at the basal surface of the cells, as single electron-dense particles (Figs. 6A, 6B, arrows) and occasionally as aggregated particles – mainly on cell membrane protrusions (Figs. 6A, 6B, arrowheads). The area of stress fibers appeared as a narrow sheet of dense arranged filaments that lined the basal cell surface.

In epithelial cancer cells of primary breast cultures, a more intense integrin alphavbeta3 immunolabeling was observed over the area of stress fibers, where mainly single gold particles (Figs. 6C, 6D, arrows) and more occasionally aggregates of them (Figs. 6C, 6D, arrowheads) were distributed. The area of stress fibers appeared wider than that of normal cells, consisting of filaments arranged in parallel and forming an angle with the substratum, indicating a well-formed actin cytoskeleton. Therefore, the ultrastructural study of integrin alphavbeta3 immunolabeling showed a colocalization of this integrin with the stress fibers at the basal surface of the cell, where focal contacts are formed. The increase of its labeling in cancer epithelial cells, in combination with the better formed actin cytoskeleton, indicates the mediation of this integrin to the cell spreading, enhancing their adhesive capacity.

### Western immunoblotting for integrin beta3 subunit and actin

**Figure 7:**
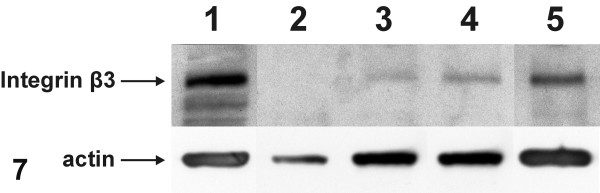
Representative Western immunoblots for integrin beta3 subunit and actin on whole cell extracts of MDA-MB-435 cell line (lane 1), normal breast tissue primary culture cells (lane 2) and primary culture cells of infiltrating breast carcinomas (grade II) (lanes 3, 4, 5). Equal amounts of protein (40 μg) were loaded from each sample and subjected to Western blot analysis. Integrin beta3 subunit was not detected in normal breast tissue cells, while differential expression of beta3 was observed in breast cancer cell samples. In the latter, a higher expression of actin was also observed in comparison with that of normal breast tissue cells.

Western immunoblotting of integrin beta3 assessed quantitatively the expression of integrin alphavbeta3 in breast cancer cells vs. normal breast cells. In parallel, the level of actin expression was examined in the same samples. Human breast carcinoma cell line MDA-MB-435 was used as positive control, since it highly expresses this integrin alphavbeta3 [24]. Equal amounts of protein (40 μg) were loaded from each sample and the expression levels of integrin beta3 subunit and of actin were studied. No detectable amounts of integrin beta3 subunit were observed in normal breast tissue primary culture cells (Fig. 7, lane 2). However, integrin beta3 subunit was expressed at various levels in breast cancer cells derived from different biopsies of infiltrating breast carcinomas with the same histological grade (grade II) (Fig. 7, lanes 3, 4, 5), confirming the heterogeneity characterizing breast cancer. In fact, one of the breast cancer samples (Fig. 7, lane 5) expressed the integrin beta3 at approximately the same high level of that of MDA-MB-435 cell line (Fig. 7, lane 1). On the other hand, actin was expressed at lower levels in normal breast cells (Fig. 7, lane 2) compared to the higher expression levels that were observed in breast cancer cells from primary cultures (Fig. 7, lanes 3, 4, 5). The latter were almost the same with that expressed in the MDA-MB-435 cell line (Fig. 7, lane 1).

## Discussion

In the present study, for the first time, integrin alphavbeta3 localization was correlated to the F-actin cytoskeleton formation in primary cell cultures of human breast cancer biopsies in comparison to normal tissue. By means of immunofluorescence, a shift of integrin alphavbeta3 localization was observed from the main cell body – particularly around the nucleus at the ventral cell surface of primary normal breast epithelial cells – to the marginal area of primary breast cancer epithelial cells. In parallel, in the latter, F-actin cytoskeleton appeared well-formed, consisting of numerous thicker stress fibers, compared to the F-actin cytoskeleton in normal epithelial cells. Furthermore, by means of electron microscopy, increased integrin alphavbeta3 immunogold localization was observed in epithelial breast cancer cells over the area of stress fibers at the basal cell surface, in comparison to normal breast epithelial cells. These findings were also verified by the higher expression of integrin beta3 subunit in primary breast cancer cells, as shown by Western immunoblotting.

Many preclinical studies have been carried out using established breast cancer cell lines, due to their easy manipulation, the infinite cell quantities and the high degree of homogeneity [32,33]. However, cell lines frequently have undergone multiple genotypic and phenotypic changes, resulting in a drift away from the biological behavior of the primary tumor of origin. On the contrary, short-term primary cell cultures of epithelial breast cancer cells, despite the culturing difficulties, consist an experimental model system with a greater biological and clinical relevance [32,34]. In the present study, primary cell cultures of breast cancer and normal tissues were developed using the explant technique, in which small fragments of minced tissue were allowed to attach onto substrate without any previous trypsinization, in order to preserve the integrin alphavbeta3 status of the cells [35]. From the adhered tissue fragments, a mixed cell population consisted of epithelial cells and stromal fibroblasts, were grown out. Although the presence of fibroblasts is considered by many investigators as "contamination" due to their high rate of proliferation, resulting in outgrowing the cancer epithelial cells, the culture conditions which were used in this study, allowed the controlled growth of a low number of fibroblast-like cells. Actually, their presence in the developed primary breast cancer cell cultures was considered essential, resembling more closely the in vivo environment of the tumor tissue, since tumor epithelial cells within the cancerous mammary gland are in direct contact with the highly activated collagenous tumor stroma, communicating with the fibroblasts and interacting with the extracellular matrix, where integrins are implicated [36,37]. Furthermore, according to recent studies, fibroblasts, except their fundamental functions, such as deposition of extracellular matrix, regulation of epithelial differentiation, regulation of inflammation [38], have also a prominent role in defining the rate and extent of cancer progression [37,39]. Hence, the model system of primary breast cancer cell culture that was developed, in the present study, retains the most closely resembling environment to the tumor tissues.

Integrins provide the physical link between the actin cytoskeleton and the extracellular matrix [25] contributing to the formation of the focal adhesion sites [40]. Specifically, integrin alphavbeta3 expression provides broad adhesive capacity of the cells, mediating the cell spreading and migration [24,41]. It behaves as an allosteric switch between a freely diffusing non-activated plasma membrane protein and an activated bivalent linker protein, binding simultaneously to extracellular ligands and cytoplasmic adaptor proteins [40]. Upon extracellular ligand binding, integrin alphavbeta3 is activated, triggering its clustering, which is required for the formation of focal adhesion sites [40,42].

In the present study, the integrin alphavbeta3 immunofluorescence in the primary epithelial cells of the normal breast tissues showed a slightly diffused pattern at the ventral surface of the cells, representing the non-clustered non-activated state of the integrin [40], freely diffused in the plasma membrane. In addition, integrin alphavbeta3 was evenly localized around the nucleus, at the main cell body of many cells. According to Cluzel et al. [40], integrin beta3 subunit clusters were observed underneath the main cell body of the mouse B16F1 melanoma cells with confocal microscope at the level of the ventral cell surface representing de novo-activated integrins, which are precursors of focal adhesions. Concerning the integrin alphavbeta3 trafficking via a "long-loop" of recycling, after its internalization, it proceeds to early endosomes, then to the perinuclear recycling compartment and finally returns to the plasma membrane [43]. Therefore, in the present study, the integrin alphavbeta3 immunofluorescence at the perinuclear area of the primary normal epithelial cells may represent either de novo accumulations of this integrin or integrins transferred to the basal cell surface after their recycling through the perinuclear compartment. Taking into account that the primary normal breast epithelial cells are not characterized by high motility, this perinuclear localization may indicate a pool of non-recruited heterodimers of integrin alphavbeta3 in the basal cell surface.

In the primary epithelial cells of the breast cancer tissues, the integrin alphavbeta3 immunofluorescence was not observed at the ventral cell surface around the nucleus, suggesting that its recycling was completed via a "short-loop", without passing through the perinuclear recycling compartment, but returning – after its internalization and proceeding to early endosomes – to the plasma membrane [43], in response to the elevated requirements of integrin alphavbeta3 availability due to the higher cellular motility. On the contrary, integrin alphavbeta3 immunofluorescence was mostly observed as bright aggregations at the cell periphery and particularly at the leading edge of the advancing lamellipodium, contributing to the adherence of the cancer cells to the substrate, forming focal adhesions and mediating their spreading and migration [24,26]. The occasional presence of integrin alphavbeta3 immunofluorescence at the rest of the main cell body in the primary breast cancer epithelial cells, as shown by the frequency distribution histogram of the immunofluorescence intensity values, may be, either, due to non-clustered non-activated integrins, where a diffused pattern is observed, or due to de novo-formed clusters of integrin, where immunofluorescent aggregations are displayed.

In parallel, in primary breast cancer epithelial cells the well-formed F-actin cytoskeleton – in comparison with that in normal counterparts – consisted by numerous thicker stress fibers, reveals the dynamic reorganization of actin filaments, reciprocating the increased integrin alphavbeta3 clustering and the formation of focal adhesion structures [30,40]. Stress fibers were observed to be oriented towards the boundary between cells, where integrin alphavbeta3 is located, but mostly towards integrin aggregations at the marginal region of the cells, as well as at the leading edge of the advancing lamellipodia, terminating at the focal adhesion structures [44,45]. Furthermore, the orientation of F-actin stress fibers to the integrin alphavbeta3 aggregations at the cell periphery may indicate the involvement of stress fibers in the translocation of this integrin also to the focal adhesion sites, as it was proved for integrin beta1 by Kawakami et al. [44].

The immunogold ultrastructural localization of the integrin alphavbeta3 in correlation with the actin cytoskeleton distribution is performed for the first time in primary breast cancer cell cultures. At the focal adhesion sites at the ventral surface of the cells, an increased integrin alphavbeta3 immunogold labeling was observed over a wider area of well-formed stress fibers arranged in parallel, compared with that of normal cells. The increased integrin alphavbeta3 immunogold labeling, combined with a well-formed actin cytoskeleton, suggests the involvement of integrin alphavbeta3 to better cellular adhesion as part of the enhanced cellular motility process [24].

The increased integrin alphavbeta3 immunolabeling at the ultrastructural level was quantified in primary breast cancer cells vs. normal ones, performing Western immunoblotting for the integrin beta3 subunit. Integrin beta3 subunit was chosen as it forms heterodimers uniquely with the v and the platelet-specific IIb alpha-chains [46]. In breast epithelial cells integrin beta3 subunit pairs exclusively with alphav to form the heterodimer alphavbeta3. Thus, the quantitation of beta3 subunit by Western immunoblotting in primary cell cultures of breast biopsies, indirectly equalizes with the quantitation of integrin alphavbeta3 expression. In primary cells of normal breast tissues, using the optimal protein load (40 μg), integrin beta3 was expressed at low no-detectable levels, which is in agreement with the no-detectable levels of integrin alphavbeta3 in MCF-7 low metastatic cell line [24]. On the contrary, in primary cells of breast cancer tissues, integrin beta3 was expressed at various levels, although the samples of breast cancer biopsies originated from the same histological type and grade, revealing the heterogeneity which characterizes the breast cancer. In one of the samples, integrin beta3 was expressed almost at the same high level, as in the highly metastatic MDA-MB-435 cell line, which was used as a positive control. On the other hand, in Western immunoblotting, the higher expression levels of actin in primary breast cancer cells compared to that in normal breast cells is consistent with the well-formed actin cytoskeleton observed in the former by immunofluorescence, suggesting responsiveness of actin cytoskeleton to the higher motility demands which are determined by the malignant potential of the breast cancer cells.

## Conclusion

In the present work, a model system of primary breast cancer cell culture was developed. Given that the most preclinical breast cancer studies are based on established cell lines which, however, may not reflect the primary tumor of origin due to genotypic changes, the developed model system of primary breast cancer cell culture consists a reliable experimental tool for the assessment of tumor cell behavior, as it is more closely related to the in vivo conditions of breast tumor. Immunofluorescence, immunogold ultrastructural localization and Western immunoblotting, showed redistribution and modulation of availability of integrin alphavbeta3 in response to the motility rate and the malignant potential of the cultured cells with concomitant F-actin cytoskeletal reorganization. Since integrin clustering and stress fiber formation consist an essential part of a mechanism inducing firm cell adhesion, our findings provide insight in the integrin alphavbeta3 inhibitors research, related to the prevention of the metastatic spread of breast cancer cells.

## Material

The material for this study consisted of 8 biopsies of infiltrating ductal breast carcinomas, surgically removed from female patients of the medical center "Prolipsis" ("Prevention"). The histopathological grading was done according to the method of Bloom and Richardson [47]. Four of the samples classified as grade I, and four as grade II. Four cases of normal breast tissue, adjacent to the cancer tissue were also included in the study for comparison. Informed consent was obtained for all medical procedures performed during the course of the patient's illness. All investigational biopsies were approved by the Athens Medical School Ethical Committee (approval no.5758/12-3-03). Histopathological grading, cell culturing and further processing of the tissue were carried out at the Laboratory of Histology and Embryology, Athens Medical School.

## Methods

### Primary cell cultures of breast tissues

Breast tissue biopsies after the operation, were immediately placed into sterilized culture medium consisting of RPMI 1640 supplemented with 10% Foetal Calf Serum (FCS), 1 mM L-glutamine, 0.5 mgr/ml gentamycin, and transported to the tissue culture facility. Small tissue fragments (2–3 mm^3^) of each biopsy were put on sterilized round glass cover slips, 13 mm in diameter (one tissue fragment/coverslip), which were already placed in plastic flasks 25 cm^2^. The cells were allowed to migrate out from tissue fragments and to grow up adhering to the glass coverslips, in the culture medium, at 37°C, 5% CO_2 _in incubator. The cell growth was complete in about 2–3 weeks.

### Scanning electron microscopy (SEM)

For SEM, cells – attached on the glass coverslips – were fixed in 2.5% glutaraldehyde in 0.05 M cacodylate-Na buffer, pH 7.4, for 30 min at 37°C and then rinsed in buffer. Dehydration was accomplished by serial immersion of the specimens for 3 min each in 25%, 50%, 70%, 95% ethanol and for 5 min in 100% ethanol twice, at room temperature (RT). The specimens were then infiltrated gradually in a mixture of amyl acetate diluted in 100% ethanol (1:2, 1:1, 2:1), 5 min each at RT and finally in 100% amyl acetate 3 times for 5 min. The specimens were then covered with one drop of hexamethyldisilazane and were dried overnight. Before the observation, they were rendered conductive by sputtering them with gold before being observed by the scanning electron microscope JEOL JSM 4500 operated at 3 kV. Digitized images were saved as high resolution *BMP files and were printed at 1000 dpi on a Xerox HP Laserjet 4250 n high resolution laser printer.

### Integrin alphavbeta3 immunofluorescence combined with F-actin phalloidin-rhodamine fluorescence

When cell culture was completed, the cells – attached onto the glass coverslips – were fixed in 3% paraformaldehyde in cytoskeleton buffer, pH 6.8, for 15 min at room temperature (RT). Each one of the coverslips was placed in a well of multiwell Petri dish and washed three times (5 min each) with cytoskeleton buffer. Cells were then incubated in primary monoclonal mouse anti-human integrin alphavbeta3 antibody LM609 (60 μg/ml) (MAB1976, Chemicon) diluted in cytoskeleton buffer, pH 6.8, containing 5% normal goat serum (NGS), overnight at 4°C, without any previous detergent permeabilization. After three washes (5 min each) with cytoskeleton buffer, cells were incubated in the secondary goat anti-mouse IgG FITC-conjugated antibody (1:300) (AP308F, Chemicon) diluted in cytoskeleton buffer, pH 6.8, containing 1% bovine serum albumin (BSA) (A7638, Sigma), for 1 h at RT. Subsequently, cells were washed three times with cytoskeleton buffer (5 min each) and permeabilized with 0.5% (vol/vol) Triton X-100 for 3 min at RT, for the visualization of filamentous actin with the application of the rhodamine-conjugated phalloidin method. After three washes with cytoskeleton buffer (5 min each), cells were incubated with rhodamine-conjugated phalloidin (3 ng/ml) (P1951, Sigma), for 1 h at 37°C. Finally, the cells were washed three times with cytoskeleton buffer and three times more with distilled water. Each glass coverslip was placed upside-down and mounted on a slide in a drop of aqua-poly mount (18606, Polysciences) antifading agent. Cells were observed with a Zeiss Axiovert S-100 inverted photomicroscope equipped with epifluorescence optics and using a 63× oil immersion objective. Photographs were recorded on Kodak 400ASA color film. The negatives were scanned in an Agfa Duoscan T2500 high resolution scanner and when required prints were obtained using a HP color Laserjet 4700 n printer.

### Image analysis morphometry

For the assessment of the relative integrin alphavbeta3 fluorescence intensities with image analysis morphometry, at the periphery and the main body of the epithelial breast cancer cells and of the epithelial breast cells of normal tissues, approximately 35 micrographs of cells of each category were taken randomly using the 63× oil immersion objective, with the same identical settings, on a Zeiss Axiovert S-100. The micrograph negatives were scanned with an Agfa DuoScan T2500 scanner at an optical resolution of 1000 ppi (pixels per inch). The digitized images were converted automatically into positive grayscale images (256 grey levels), stored as tiff files and calibrated before use.

The image analysis was carried out using the Image-Pro Plus, version 3.0, software for Windows (Media Cybernetics, Silver Spring, MD, USA) on a Pentium IV, 2.4 GHz, workstation. The estimation of the relative integrin alphavbeta3 densities was made by measuring the integrin fluorescence intensities per defined cellular areas at the cell periphery and the main cellular body with specially developed macros, as follows: a) calibration of the intensity curve per image b) interactive drawing of the cell periphery as it is defined by the outer limit of the cell surface and 1–5 μm inwards, as this distance corresponds to the usual breadth of a lamellipodium [29], (c) interactive drawing of the main body of the cell as it is defined by the rest cellular area, excluding the nuclear area. All adjustments, including programming and calibrations, were made by a certified (factory approved) expert programmer.

The values of the relative integrin alphavbeta3 fluorescence intensities per cell were automatically recorded in a sheet of Excel. Their statistical assessment between normal and cancer cells was performed using the "frequency distribution" test. The values were divided into 8 classes. The distribution of frequency of values which corresponds to the cell periphery and the main cellular area is displayed comparatively in each histogram concerning the normal and cancer epithelial cells.

### Transmission electron microscopy

#### Tissue preparation

For electron microscopy, cells allowed to migrate from biopsy tissue pieces and to adhere on the plain plastic bottom of the culture flask for 2–3 weeks. They were then fixed in 2.5% glutaraldehyde made up in phosphate buffer saline (PBS) 0.01 M, pH 7.4, for 15 min at RT, post-fixed in 1% aqueous OsO_4 _for 30 min at 4°C, isolated according to Kouloukoussa et al. [48] and finally embedded in a mixture of epoxy resins. Ultrathin (80–90 nm thick) sections were cut with a Diatome diamond knife on an Leica Ultracut R ultramicrotome and were collected onto 200 mesh uncoated nickel grids.

#### Electron immunocytochemistry

Immunogold labeling of integrin alphavbeta3 was carried out as described previously [49], modified for single labeling. Briefly, all incubations were carried out by floating the grids on drops of solutions at RT, except for the primary antibody step, using the multiwell Terasaki plates as humid chambers. Before use, all solutions were micro-filtered with millipore filters (0.2 μm pore size) fitted to a 5 ml syringe. Grid washing was carried out by floating them on drops of the washing solutions with simultaneous gentle agitation on a mechanical agitator. Before the immunolabeling procedure, the nickel grids with the osmicated epoxy-resin sections were first incubated on drops of 8% H_2_O_2_, in a humid chamber for 8 min and then on drops of saturated aqueous solution of NaIO_4 _[50], pH 3.8 for 30 min at RT, in order to remove osmium from the superficial regions of the thin sections to be labeled. Between these two incubations, thorough rinsing with distilled water before immunolabeling was carried out.

#### Immunogold labeling of integrin alphavbeta3

Grids were first incubated for 4 min (four changes, 1 min each) on drops of 0.05 M Tris/HCl buffer, pH 7.4, and then on blocking buffer [0.05 M Tris/HCl, pH 7.4+0.2% BSA+0.1% coldwater fish gelatin+5% NGS+0.1% Tween-20] for 30 min. Grids were then incubated in the primary monoclonal mouse anti-human integrin alphavbeta3 (10 μg/ml) antibody diluted in 0.05 M Tris/HCl buffer, pH 7.4, containing 1% BSA, overnight at 4°C. Control sections were incubated in the absence of primary antibody. The grids were rinsed, with agitation, in five changes of 0.05 M Tris/HCl, pH 7.4, for 2 min each time, and then they were washed, with agitation, consecutively in five changes (1 min each) of 0.05 M Tris/HCl, pH 7.4 containing 0.1% Tween-20 (solution I), three changes (1 min each) of 0.05 M Tris/HCl, pH 7.2 containing 0.2% BSA and 0.1% Tween-20 (solution II), one change for 5 min of 0.05 M Tris/HCl, pH 8.2 containing 1% BSA and 0.1% Tween-20 (solution III). The grids were incubated for 1 h at RT in the secondary colloidal gold antibody [goat anti-mouse IgG conjugated to 15 nm gold particles (1:20) (25133, EMS)] diluted in solution III and they were washed with agitation in 3 changes (1 min each) of solution II, five changes (1 min each) of solution I and five changes (1 min each) of distilled water. Finally, the grids were dried and counterstained with saturated ethanolic uranyl acetate and lead citrate. Sections were viewed in a Zeiss EM 900 electron microscope, at 80 kV accelerating voltage, with an objective aperture of 30 μm. The electron micrographs were recorded on Kodak Electron Image film SO-163 (gelatin plates, 8 cm × 10 cm, Code No: 1679257). The negatives were scanned with an Agfa DuoScan T2500 scanner using optical resolution of 1000 ppi and stored as tiff files. When required, the digitized images were printed at 1200 dpi on a HP laserjet 4250 n high resolution laser printer.

### Western immunoblotting of beta3 integrin subunit

Breast cancer, as well as normal cells from primary cultures were harvested by culture flasks by brief treatment with 0.05% Trypsin-0.53 mM EDTA (GIBCO-Invitrogen), pelleted with centrifugation and lysed in modified RIPA buffer (150 mM NaCl, 50 mM Tris-HCl pH 8.0, 0.1% SDS, 1% sodium deoxycholate, 1% NP-40, 1% Triton) containing 10 μM PMSF and 1:100 Protease inhibitors cocktail (SERVA). For homogenization, cells were left on ice for 1 h while pippeting 4–5 times every 10 min. Cell debris was removed by centrifugation at 15,000 g for 20 min and supernatant was stored. Protein concentration was determined with the BCA method (PIERCE).

After several trials, the optimum amount of loaded protein was determined at 40 μg. Therefore, equal amounts of protein (40 μg) were loaded and resolved on 10% SDS-PAGE, after addition of 4× Laemli buffer (200 mM Tris-HCl pH 6.8, 8% SDS, 40% Glycerol, 20% 2-mercaptoethanol, 0.8% Bromophenol blue). In each gel, 10 μL of prestained Molecular Weight Marker (PageRuler, Fermentas) was loaded. Gels were electroblotted on nitrocellulose membranes (0.45 μm pore size, Amersham) at 20 V overnight and membranes were separated in two parts according to the bands of the Molecular Weight Marker. Membranes were then blocked with 5% nonfat dry milk in TBS, containing 0.05% Tween-20, 0.01% azide, for 2 h at RT. The parts of the membranes containing proteins from 75 to 175 kDa were incubated overnight at 4°C with primary antibody rabbit anti-human integrin beta3 (1:2,000) (AB1932, Chemicon) and the parts of the membranes containing proteins from 20 to 75 kDa were incubated overnight at 4°C with the mouse anti-human actin antibody (1:2,000) (MAB1501, Chemicon). The next day the membranes were washed with TBS, containing 0.05% Tween-20 and 0.01% azide (4 × 15 min). The parts of the membranes incubated with anti-human beta3 were subsequently incubated with secondary antibody goat anti-rabbit IgG-HRP conjugated (1:5,000) (AP307P, Chemicon) and the parts of the membrane incubated with anti-actin were subsequently incubated with rabbit anti-mouse IgG-HRP conjugated (1:3,000) (P0161, Dako), for 1 h at RT. After washing with TBS containing 0.05% Tween-20 and 0.01% azide (4 × 10 min), peroxidase activity was visualized with ECL Plus reagent (Amersham).

## Abbreviations

PI(4,5)P2 phosphoinositol-4,5-biphosphate

FCS foetal calf serum

RT room temperature

NGS normal goat serum

PBS phosphate buffer saline

BSA bovine serum albumin

TBS tris buffer saline

HRP horseradish peroxidase

## Competing interests

The author(s) declare that they have no competing interests.

## Authors' contributions

SH is the principal investigator employed by a post-doctoral fellowship. She made all necessary lab work, cell culturing, morphometry, electron microscopy and photography. MK is a cell culture specialist who coordinated primary culture establishment, growth and maintenance. KA worked on the fluorescent experiments and photography. YD was responsible for cell isolation and Western immunoblotting. LDA is a resident pathologist who examined tissue biopsies. NG is a pathologist, responsible for tissue biopsy delivery and grading. DV is a pathologist responsible for handling tissue biopsies and isolation of cancer lesions. IVB is a cell culture expert who acted as a consultant. VAM is a human genetics specialist who worked on Western immunoblottings and electrophoreses. SDV is a senior surgeon of the hospital responsible for routine surgeries. EZK was involved in designing and performing western immunoblotting experiments. CK is Head of the Department and pathologist who participated in experiment design and helped to draft the manuscript. EM worked as an electron microscopy specialist for the examination of the samples under the electron microscope and coordinated the research group.

## References

[B1] Plow EF, Haas TA, Zhang L, Loftus J, Smith JW (2000). Ligand binding to integrins. J Biol Chem.

[B2] Albelda SM, Buck CA (1990). Integrins and other cell adhesion molecules. FASEB J.

[B3] Rudolph R, Cheresh D (1990). Cell adhesion mechanisms and their potential impact on wound healing and tumor control. Clin Plast Surg.

[B4] Heino J (1993). Integrin-type extracellular matrix receptors in cancer and inflammation. Ann Med.

[B5] Felding-Habermann B, Cheresh DA (1993). Vitronectin and its receptors. Curr Opin Cell Biol.

[B6] Giancotti FG, Ruoslahti E (1999). Integrin signaling. Science.

[B7] Kerr JS, Slee AM, Mousa SA (2002). The alpha v integrin antagonists as novel anticancer agents: an update. Expert Opin Investig Drugs.

[B8] Morozevich GE, Kozlova NI, Chubukina AN, Berman AE (2003). Role of integrin alphavbeta3 in substrate-dependent apoptosis of human intestinal carcinoma cells. Biochemistry (Mosc).

[B9] Zutter MM, Sun H, Santoro SA (1998). Altered integrin expression and the malignant phenotype: the contribution of multiple integrated integrin receptors. J Mammary Gland Biol Neoplasia.

[B10] Mizejewski GJ (1999). Role of integrins in cancer: survey of expression patterns. Proc Soc Exp Biol Med.

[B11] Sloan EK, Pouliot N, Stanley KL, Chia J, Moseley JM, Hards DK, Anderson RL (2006). Tumor-specific expression of alphavbeta3 integrin promotes spontaneous metastasis of breast cancer to bone. Breast Cancer Res.

[B12] Fawcett J, Harris AL (1992). Cell adhesion molecules and cancer. Curr Opin Oncol.

[B13] Albelda SM, Mette SA, Elder DE, Stewart R, Damjanovich L, Herlyn M, Buck CA (1990). Integrin distribution in malignant melanoma: association of the beta 3 subunit with tumor progression. Cancer Res.

[B14] Dang D, Bamburg JR, Ramos DM (2006). Alphavbeta3 integrin and cofilin modulate K1735 melanoma cell invasion. Exp Cell Res.

[B15] Gingras MC, Roussel E, Bruner JM, Branch CD, Moser RP (1995). Comparison of cell adhesion molecule expression between glioblastoma multiforme and autologous normal brain tissue. J Neuroimmunol.

[B16] Hapke S, Kessler H, Luber B, Benge A, Hutzler P, Hofler H, Schmitt M, Reuning U (2003). Ovarian cancer cell proliferation and motility is induced by engagement of integrin alpha(v)beta3/vitronectin interaction. Biol Chem.

[B17] Cooper CR, Chay CH, Pienta KJ (2002). The role of alpha(v)beta(3) in prostate cancer progression. Neoplasia.

[B18] Harms JF, Welch DR, Samant RS, Shevde LA, Miele ME, Babu GR, Goldberg SF, Gilman VR, Sosnowski DM, Campo DA, Gay CV, Budgeon LR, Mercer R, Jewell J, Mastro AM, Donahue HJ, Erin N, Debies MT, Meehan WJ, Jones AL, Mbalaviele G, Nickols A, Christensen ND, Melly R, Beck LN, Kent J, Rader RK, Kotyk JJ, Pagel MD, Westlin WF, Griggs DW (2004). A small molecule antagonist of the alpha(v)beta3 integrin suppresses MDA-MB-435 skeletal metastasis. Clin Exp Metastasis.

[B19] Vellon L, Menendez JA, Lupu R (2006). A bidirectional "alpha(v)beta(3) integrin-ERK1/ERK2 MAPK" connection regulates the proliferation of breast cancer cells. Mol Carcinog.

[B20] Liapis H, Flath A, Kitazawa S (1996). Integrin alpha V beta 3 expression by bone-residing breast cancer metastases. Diagn Mol Pathol.

[B21] Rolli M, Fransvea E, Pilch J, Saven A, Felding-Habermann B (2003). Activated integrin alphavbeta3 cooperates with metalloproteinase MMP-9 in regulating migration of metastatic breast cancer cells. Proc Natl Acad Sci USA.

[B22] Felding-Habermann B, O'Toole TE, Smith JW, Fransvea E, Ruggeri ZM, Ginsberg MH, Hughes PE, Pampori N, Shattil SJ, Saven A, Mueller BM (2001). Integrin activation controls metastasis in human breast cancer. Proc Natl Acad Sci USA.

[B23] Gomes N, Vassy J, Lebos C, Arbeille B, Legrand C, Fauvel-Lafeve F (2004). Breast adenocarcinoma cell adhesion to the vascular subendothelium in whole blood and under flow conditions: effects of alphavbeta3 and alphaIIbbeta3 antagonists. Clin Exp Metastasis.

[B24] Wong NC, Mueller BM, Barbas CF, Ruminski P, Quaranta V, Lin EC, Smith JW (1998). alphav integrins mediate adhesion and migration of breast carcinoma cell lines. Clin Exp Metastasis.

[B25] Hynes RO (1992). Integrins: versatility, modulation, and signaling in cell adhesion. Cell.

[B26] Ballestrem C, Hinz B, Imhof BA, Wehrle-Haller B (2001). Marching at the front and dragging behind: differential alphaVbeta3-integrin turnover regulates focal adhesion behavior. J Cell Biol.

[B27] Ballestrem C, Wehrle-Haller B, Imhof BA (1998). Actin dynamics in living mammalian cells. J Cell Sci.

[B28] Horwitz AR, Parsons JT (1999). Cell migration – movin' on. Science.

[B29] Small JV, Stradal T, Vignal E, Rottner K (2002). The lamellipodium: where motility begins. Trends Cell Biol.

[B30] Petit V, Thiery JP (2000). Focal adhesions: structure and dynamics. Biol Cell.

[B31] Hynes RO (2002). Integrins: bidirectional, allosteric signaling machines. Cell.

[B32] Burdall SE, Hanby AM, Lansdown MRJ, Speirs V (2003). Breast cancer cell lines: friend or foe?. Breast Cancer Res.

[B33] Pettengill OS, Lewko WM (1989). Cell culture of human metastatic breast carcinomas: a review. Mol Biother.

[B34] Speirs V, Green AR, Walton DS, Kerin MJ, Fox JN, Carleton PJ, Desai SB, Atkin SL (1998). Short-term primary culture of epithelial cells derived from human breast tumours. Br J Cancer.

[B35] Brown MA, Wallace CS, Anamelechi CC, Clermont E, Reichert WM, Truskey GA (2007). The use of mild trypsinization conditions in the detachment of endothelial cells to promote subsequent endothelialization on synthetic surfaces. Biomaterials.

[B36] Kalluri R, Zeisberg M (2006). Fibroblasts in cancer. Nat Rev Cancer.

[B37] Orimo A, Weinberg RA (2006). Stromal fibroblasts in cancer: a novel tumor-promoting cell type. Cell Cycle.

[B38] Parsonage G, Filer AD, Haworth O, Nash GB, Rainger GE, Salmon M, Buckley CD (2005). A stromal address code defined by fibroblasts. Trends Immunol.

[B39] Elenbaas B, Weinberg RA (2001). Heterotypic signaling between epithelial tumor cells and fibroblasts in carcinoma formation. Exp Cell Res.

[B40] Cluzel C, Saltel F, Lussi J, Paulhe F, Imhof BA (2005). The mechanisms and dynamics of alphavbeta3 integrin clustering in living cells. J Cell Biol.

[B41] Wayner EA, Orlando RA, Cheresh DA (1991). Integrins alpha v beta 3 and alpha v beta 5 contribute to cell attachment to vitronectin but differentially distribute on the cell surface. J Cell Biol.

[B42] Miyamoto S, Teramoto H, Coso OA, Gutkind JS, Burbelo PD, Akiyama SK, Yamada KM (1995). Integrin function: molecular hierarchies of cytoskeletal and signaling molecules. J Cell Biol.

[B43] Caswell PT, Norman JC (2006). Integrin trafficking and the control of cell migration. Traffic.

[B44] Kawakami K, Tatsumi H, Sokabe M (2001). Dynamics of integrin clustering at focal contacts of endothelial cells studied by multimode imaging microscopy. J Cell Sci.

[B45] Kumar CC (1998). Signaling by integrin receptors. Oncogene.

[B46] Tucker GC (2006). Integrins: Molecular targets in cancer therapy. Curr Oncol Rep.

[B47] Bloom HJG, Richardson HH (1957). Histological grading and prognosis in breast cancer. A study of 1409 cases of which 359 have been followed for fifteen years. Brit J Cancer.

[B48] Kouloukoussa M, Panagopoulou E, Marinos E (1999). The in vitro effect of the tumor promoter 12-O-tetradecanoylphorbol-13-acetate on Sertoli cell morphology. Cancer Detect Prev.

[B49] Havaki S, Dafni U, Sotiropoulou C, Voloudakis-Baltatzis I, Goutas N, Vassilaros SD, Athanasiou E, Arvanitis DL, Kittas C, Marinos E (2003). Ultrastructural immunostaining of infiltrating ductal breast carcinomas with the monoclonal antibody H: a comparative study with cytokeratin 8. Ultrastruct Pathol.

[B50] Bendayan M, Zollinger M (1983). Ultrastructural localization of antigenic sites on osmium-fixed tissues applying the protein A-gold technique. J Histochem Cytochem.

